# Trachelorraphy

**Published:** 1893-06

**Authors:** G. J. Starnes

**Affiliations:** San Antonio, Texas


### DANIEL'S TEXAS MEDICAL JOURNAL.

ESTABLISHED JULY, 1885.

PUBLISHED MONTHLY.—SUBSCRIPTION $2.00 A YEAR.

Vol. VIII. AUSTIN, JUNE, 1893. No. 12.

### Original Contributions.

For Daniel's Texas Medical Journal.

### TRACHELORRAPHY.

G. J. STARNES, M. D., SAN ANTONIO, TEXAS.

[Read before the Lone Star Medical Association, at Galveston, Texas.]

THE subject under consideration has been a topic of so many discussions and furnished a thesis for so many writers within the last decade, that I feel myself wholly incompetent to interest you with a paper. However, I shall endeavor to present the treatment and the principal features of the operation, together with a few cases operated upon by myself, thereby hoping to have you, in the words of Munde, "see the string on which my beads are strung."

We mean by trachelorraphy a suture, a term used to describe the operation for the repair of the lacerated cervix, better known in this country as "Emmet's operation." Dr. Emmet, of New York, first conceived the idea in 1862, and later, in 1868, read a paper in support of his views before the New York County Medical Society. I shall set forth, first, the treatment preparatory to an operation, and also, the treatment of the conservative, which sometimes relieves the existing symptoms and often promotes a cure. The classes of lacerations are generally three: unilateral, bi-lateral and stellate or star shaped, either of which may be diagnosed by the touch.

The initiatory treatment for a recent laceration should be: vaginal douches as hot as can be borne; this serves a two-fold object, namely that of cleanliness and depleting the engorged capillaries, thereby reducing weight and congestion in the parts. The good effects of the douche may be increased by the addition of antiseptics to the lotion above named. The results of the douche are about the same, regardless of the class to which the laceration belongs. The antiseptic lotion, besides its cleansing properties, prevents the absorption of septic material, thereby warding off para-metritis and peri-metritis, peritonitis, cellulitis, metritis and a host of kindred ailments, heir to the same cause. Besides this, the antiseptic lotion has the effect of husbanding the parts--bringing the rent surfaces into apposition and often causing union from the first intention. This constitutes the bulk of treatment required in an ordinary case of recent laceration, and a great many cases go on to healthy recovery without any treatment whatever.

Where the physician finds a case of laceration which has for days and months been a source of irritation, the above treatment is wholly inadequate, but forms a splendid adjuvant to the field of other remedies that must be brought to bear before a cure can be oblained, if obtained at all. It should be borne in mind that the antiseptic douche must be used first, last and all the time.

Frequently, on examination, a crop of little cystic eminences will be found projecting from the os. These may be destroyed by puncturing with Battle's scarifier as a bistoury, allowing the watery contents to escape, and afterwards painting the parts with Churchill's iodine, cauterising with pyroligneous acid, fuming nitric acid or any cautery that will destroy the little glands that produce them; and this treatment continued at intervals from two to three times per week until they cease to appear. When a denuded surface presents itself upon the os and lacerated portion and a thin mucus discharge of an acid nature is kept up, it is plain that the acid discharge prevents healing; then alkaline washes are indicated, to neutralize the acid: Sodium phosphate, sodium borate, bismuth in solution or sprinkled dry over the parts, or a tampon, saturated with a solution or sprinkled with the powders, may be introduced and forced up well under the posterior lip of the os and kept intact for at least from twenty-four to forty-eight hours, and always followed by the hot douche, when removed. This course persevered in twice per week for four or five weeks, ought to be followed by favorable results.

In other patients there will be found protruding from the cervix a thick, viscid, tenacious, discharge, which is only removed with considerable effort. Before attempting to make applications this mucus should be removed and the parts thoroughly cleansed. This may be accomplished by taking a piece of cotton in Sim's or Emmet's dressing forceps (any other may do as well but I have found these to answer my purpose best), passing it well into the cervix, and then, by twisting the mucus into the meshes or fibres of cotton, detach it. Frequently the addition of a little alcohol or carbolic acid will coagulate the mucus; it then can be seized with the forceps and removed immediately. After thorough cleansing, the parts should be cauterized with a 40 per cent. solution of carbolic acid, Churchill's iodine, chrysophanic acid, or blue vitriol, so applied as to come in direct contact with the open mouths of the little diseased glands that emit the discharge. A little absorbent cotton saturated with either of these remedies (except the blue vitriol) held with the dressing forceps may be applied over the parts. A single piece of the crude vitriol may be filed or ground to convenient shape and then used in the same manner as lunar caustic. The most popular way of treating the internal uterine mucous membrane is Sims'. This is an ordinary sound, with a canula attached to the end and made just the curvature of the cervix; this canula is uniformly perforated; being filled with the desired preparation, introduced into the cervix, then pressure upon the piston forces the menstruum out over the surface of the mucous membrane, distributing prettv nearly equal over all parts. This treatment having been effected, then a tampon of cotton wool, lamb's wool or non-absorbent cotton, saturated with glycerine, raw glycerole, pinus canadensis, or glycerole of tannin should be introduced and forced up well against the os, and allowed to remain there from twelve to thirty-six hours. After removing, the hot douche should be used three or four times per day, the patient remaining in the dorsal decubitus, hips elevated and shoulders lowered, retaining this position for about twenty or thirty minutes, after the douche has been used, so as to allow the lotion to come in contact with all the mucous folds of the vagina and os. In a goodly number of these patients will be found old cicatrices, infiltration of foreign tissue, adenoid tissue stasis, more or less superinduced by engorgement and increased weight, congestion and adhesions, all of which may be more or less relieved by painting with iodine and the use of tampons, as above mentioned. As complete and elaborate as the above course of treatment may appear, one will sometimes be astonished to find that some obstinate cases fail to be benefited. Then a second and more pleasing astonishment may be enjoyed by employing a little strategem, after the following manner:

Take a little lamb's wool, saturated with glycerole of tannin, and bind around the end of a tupela or sea tangle tent, then force the tent well into the cervix until the os and lamb's wool are brought in apposition, fitting squarely against each other. The os drops down upon the floor of the pelvis, the tampon and os are held in position by the weight of the uterus and stem of the tent, fitting like a tenon in a mortise, holds the parts in contact any length of time. This, gentlemen, is purely original, but none the less valuable in the treatment of an inflamed, painful and sometimes ulcerated os uteri, and of all the applications in my experience is most to be relied upon in the majority of chronic, obstinate inflammations and ulcerations.

### INDICATIONS FOR AN OPERATION.

When the above treatment has been patiently and persistently employed, and there still exists pains in the loins, lower part of the abdomen, small part of the back, occasional sick stomach without other cause, leucorrhea and general lassitude, it is then plain to reason that an operation is necessarily imperative. We scarcely ever find all of the enumerated symptoms existing in any one individual at the same time, but any or a sufficient number will justify an operation, when no other cause for the disturbance can be cited.

### OBJECT OF AN OPERATION.

The object in the operation is to aid in overcoming the subinvolution, areolar hyperplasia healing the rent, checking leucorrhea, breaking up sterility, and relieving the otherwise exhausted and depressed condition of the system, caused by the laceration.

### OPERATION.

Everything being in readiness, the patient is placed in the supine position, upon a table or bed, knees flexed. We may mention here that an anæsthetic is scarcely ever indicated, as very little pain is experienced, a Sims' speculum introduced and the uterus seized with a pair of volsellum forceps and drawn down within an easy reach of the surgeon's scissors or lance. Most operators prefer Skene's hawk-bill scissors for paring the rent surfaces; it having two advantages over the lance and one over the ordinary surgeons' scissors. Great care should be taken in cutting away the hardened and cicatrized tissue, as you will be surprised to find after a sufficient time has elapsed for union to take place, that an examination will show that the smallest remains of an old scar or burrowing tissue has prevented it. Having cut away sufficiently all unhealed tissue, we proceed to stitch; for this purpose the small curved Armstrong needle has answered my purpose best. This needle is made broadest in its anterio-posterior diameter, and when pressure is made upon the suture, the opening made by the needle is closed, or the parts are drawn more closely together, thereby causing it to heal more readily, whereas in the ordinary surgeon's needle, made broadest from side to side or lateral diameter, the opening made by the needle is made wide and union is longer taking place. When Jackson's angular needle for suturing the cervix was introduced, I thought it was the coveted boon, but a few trials has convinced me that it was not.

### THREAD USED IN SUTURING.

For this operation some prefer catgut, some silkworm gut, some silk, and others silver ligatures. I have so far obtained the best results from the use of silver; this is not so easily removed as the silk, the catgut and silkworm gut, which being absorbed, gives them a decided preference over the silver, —naturally enough the question is asked: why do you prefer the silver?—it being bunglesome and hard to remove on account of the difficulty arising on attempting to introduce the speculum to remove the stitches. The advantages of the silver are these; when the parts are properly pared and the sutures are twisted they are held in perfect apposition and union takes place from the first intention; whereas, in the use of silk and catgut there is sometimes a lapping over or slipping or tucking under of edges, which prevents union from taking place, and frequently, you find yourself in need of making a second operation, which renders some embarrassment on the part of the patient, gives room to doubt your knowledge or skill to master the situation. Most operators direct that in sewing a bi-lateral laceration the stitches be taken alternately. I have found it most convenient that when you have the parts in position to finish one side and then undertake the other; for the constant freeing and adjusting of the tenaculum increases the number of wounds in the cervix, prolongs the operation and pain, as well as the danger, in case your patient is anæsthetized, while the results are no better than taking each in its turn and finishing it, before removing the forceps and tenaculum. You will find that the books direct that in taking your stitches you will reach back about one-fourth of an inch in inserting the needle. The fourth of an inch on two sides, and that back and front upon the small organ under consideration and in the space allotted to the operation, is not as practicable in practice as in theory, and a little experience will show that an eighth or a sixth of an inch is about all that reason will justify. In the stellate or star shaped laceration we should convert it into a bi-lateral, by cutting away the smallest projection, thereby forming one posterior and one anterior lip and proceed as before to operate. There is another class of lacerations, to which name has not, as yet been given. This laceration presents a denuded anterior or posterior lip rolled upon itself and causing a regular ectropion, as the case may be. In this class of lacerations, the paring should be carried far enough back on either side to give sufficient room to cut away all the denuded surface; otherwise you fail to get the desired results. The operation finished, all the clots removed, and the vagina thoroughly cleaned by washing with a 1-40 solution of carbolic acid or a 1-3000 bi-chloride solution, a sponge sprinkled with iodoform and placed against the os, and the uterus forced well into its normal position, then a narrow strip of iodoform gauze passed into the vagina until it comes in contact with the sponge to form a partial drainage. Then a dressing of absorbent cotton should pretty well cover the external parts, and be kept there from three to five days, when the dressing and stitching should be removed, the bowels emptied, and the patient required to remain in bed for five or six days longer. During the time the dressing is kept up, the bladder should be emptied from day to day, with the catheter.

OPERATIONS.

No. 1. Mrs. B., aged 37 years, mother of three children, youngest three years.

*Symptoms:* Headache, pain in back, loins, lower portion of abdomen, leucorrhea, sick stomach, pains over the pericardial region, with general lassitude. Laceration, bi-lateral. This patient presented herself in March, 1887, and was operated upon in April of the same year. I was assisted in the operation by Dr. Burchett, of Memphis, Tenn.

*Results:* Before the operation patient was able to do about one-fourth of the work of the healthy woman; but for the last two years, she has been doing all of the cooking for a large family' going up and down stairs a dozen or more times a day, a thing which she had not been able to do foı five years.

No. 2. Mrs. H., aged 21 years, mother of one child; able to do about three-fourths of the work of the healthy female. At present perfectly healthy and stout and able to do more than the average female. This operation was made in April, 1889. Assisted by Dr. Kennedy, of San Antonio.

No. 3. Mrs. S., aged 28 years, mother of one child.

*Symptoms:* Poor digestion, constant cough; pain in small of back and lower abdomen, occasional sick stomach, headache and general malaise, able to do about one-half the work of the average female. Laceration bi-lateral. I was assisted in this operation by Dr. Townsend, of Victoria. This patient remained in my care only about a month after the operation, and I was not able to learn how much she was benefited by the operation, only as she stated through letters. She claimed to be considerably relieved. But in that one month there was improvement in the cough, the appetite, the strength and the sick stomach.

No. 4. Mrs. F., aged 20 years, mother of one child, the child aged 3 years; able to do about one-half of the work of the average female, symptoms about the same as those usually present in such cases. Laceration, an ectropion, assisted by Dr. Kingsley, of San Antonio. The operation was performed September 28, 1889. The patient became pregnant October 18th, of the same year.

No. 5. Mrs. J., aged 22 years, mother of one child; patient in bed, laceration bi-lateral, rectocele, rent complete into the rectum, patient unable to retain fœces, operation performed in December, assisted by Drs. Johns and Kingsley of San Antonio.

*Results:* The patient has been able since to follow her occupation of washing and ironing.

